# Hypotheses in Medicine

**Published:** 1846-04

**Authors:** 


### HYPOTHESES IN MEDICINE.

It is the fashion in our day to condemn hypothesis in medicine, and insist upon facts, as all in all. But have we, in reality, discarded hypothesis? Is there any thinking man who has not his hypothesis? A very distinguished statesman, who is also a farmer, once remarked to us that, although he was distrustful of theories, he had a theory that "dun mares produced larger mule colts than those of any other color." This was his hypothesis, founded on a certain number of observations, and whether true or not, observation must decide. Sir Isaac Newton, driven by the plague from London, and sitting in a garden, fell into a train of speculation on the power of gravity, the result of which was a conjecture that the same force which brought the falling apple to the ground reached to the moon, and kept her in her orbit. This was his hypothesis, distrusted long by himself, and not rendered certain by calculations until it had occupied his great mind for sixteen years. And so, had not Columbus his hypothesis when he set sail in search of the new world? Lavoisier conjectured that soda and potash were, at bottom, metals; and this became the hypothesis of Davy, who made it something more by his famous feat with the galvanic battery. Newton seeing that the diamond, like unctuous bodies, refracted the rays of light powerfully, conceived the thought that, like those bodies, diamond contained something which was combustible; and Lavoisier embracing the hypothesis converted it into positive knowledge by the experiment of burning the precious gem in oxygen gas. But are hypotheses native to great minds alone? Do we not all daily act upon them?

The practitioner is called to the bed-side of a patient; he notes the phenomena of his case, and concludes, after the best investigation he can give it, that it is chronic hepatitis; and upon this hypothesis he bases his therapeutics. If the result prove favorable to his hypothesis he is strengthened in the conviction of its truth; and if he have a number of patients with a similar aggregate of symptoms, who recover under the same treatment, he becomes at last wedded to his hypothesis.

But this is not what is generally understood by hypothesis.—"Whatever is not deduced from the phenomena, is to be termed hythesis;" these are the words of Newton; and in this sense, it is of hypothesis as of treason—

"Treason does never prosper—what's the reason?
Why, when it prospers none dare call it treason."

These lines are quoted by Dr. Bigelow in an address delivered before the Boylston Medical Society of Harvard University, which we have read with unusual pleasure. The subject of the address is "Medical Science and Medical Art," and among the topics handled by the writer with much originality is the one upon which we have bestowed the few foregoing remarks. Dr. Bigelow holds that many, if not all great discoveries in science, like those of Newton, Columbus, and Davy, 'have been preconceived before they were demonstrated.' The perversion of this is the framing of an hypothesis, and then bending facts to sustain it. This is an abuse of facts and of reason. Facts, as he correctly observes, "are to test, to prove or disprove, not to confirm theory." And, as he continues, "the error of former science, the error of recent medicine, was not that hypotheses were made, but that they were constructed to be proved; that truth was warped and misapplied; that facts were prostituted in the cause of ignorance and prejudice." Cullen framed his theory of fever, and from that time forth held all those "facts" to be "false" which did not conform to it. Hahnemann saw in medical experience a few instances giving countenance to the law—*similia similibus curantur*, and homœopathy became his hypothesis, to which all facts were made to bend. Many more cases of the same sort might easily be cited.

Assuredly facts are not to be undervalued, for a large proportion of medical science consists of facts wholly unexplained. But we agree with Dr. Bigelow, that "facts are not the end of science;" and that "medical discovery has generally been the work of individuals, who had their hypotheses and have analyzed facts in some especial relations, and not of societies, who have collected them indiscriminately." It was upon this path that Jenner marched in his great discovery; and albeit, slowly, and with opposition, and sneers enough from his companions, for they threatened to expel him from their medical society, if he continued to harass them with the disgusting subject of vaccination.

